# Challenges following CRS and HIPEC surgery in cancer patients with peritoneal metastasis: a comprehensive review of clinical outcomes

**DOI:** 10.3389/fsurg.2024.1498529

**Published:** 2024-12-02

**Authors:** Mehdi Karimi, Niyousha Shirsalimi, Eshagh Sedighi

**Affiliations:** ^1^ Faculty of Medicine, Bogomolets National Medical University (NMU), Kyiv, Ukraine; ^2^ Faculty of Medicine, Hamadan University of Medical Science (UMSHA), Hamadan, Iran; ^3^ Department of Veterinary Medicine, Islamic Azad University Branch of Urmia, Urmia, Iran

**Keywords:** cytoreductive surgery, hyperthermic intraperitoneal chemotherapy, chemotherapy, peritoneal metastasis, surgical oncology, surgery, Malignancy, cancer

## Abstract

Cytoreductive Surgery (CRS) and Hyperthermic Intraperitoneal Chemotherapy (HIPEC) are a pair of relatively modern therapeutic surgical methods in advanced cancerous patients with peritoneal metastasis (PM). The goal of CRS + HIPEC is treatment or to improve survival outcomes, which are linked to high morbidity side effects and complications, even with their possible advantages. Surgical-related, chemotherapy-related, anesthetic-related, gastrointestinal, organs and systemic complications are the categories into which complications are separated according to frequency, risk factors, and effect on patient outcomes. In this narrative review of the literature, the side effects and complications of HIPEC + CRS in cancer patients with PM are examined. The present knowledge on the incidence, frequency, kinds, and risk factors of acute complications following CRS + HIPEC is summarized in this study. This review emphasizes the need for careful patient selection criteria, precise surgical technique, and thorough intraoperative care to reduce or manage these risks. Moreover, it highlights the need for interdisciplinary collaboration in treating these patients. This study aims to know these complications, improve clinical practice, and guide future studies to increase the safety and efficacy of CRS + HIPEC in treating metastatic colorectal cancer.

## Graphical Abstract

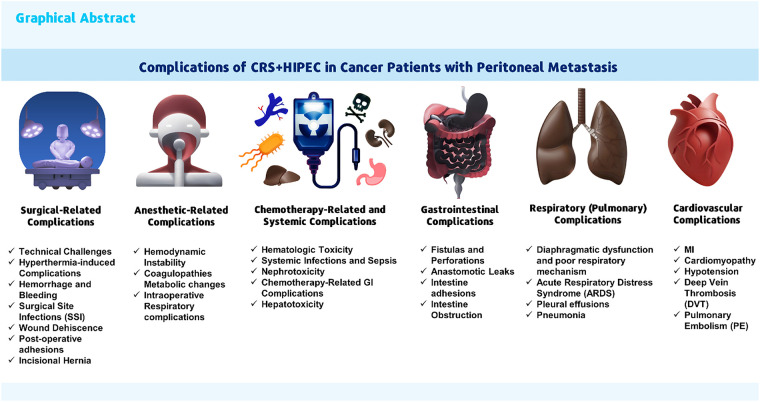

## 1 Introduction

Cytoreductive surgery (CRS) and hyperthermic intraperitoneal chemotherapy (HIPEC) are paired with modern surgical therapeutic methods to treat metastatic cancer, which is spread to the abdominal cavity and its organ and structure. CRS + HIPEC is aimed at improving survival in patients with peritoneal metastasis (PM) (1). PM, a typical progression in various abdominal cancers, significantly diminishes patient prognosis and quality-of-life (QoL) (2, 3). Traditional systemic therapies often fail to achieve sufficient therapeutic concentrations within the peritoneal cavity, necessitating alternative strategies. CRS + HIPEC has emerged as a pivotal treatment modality, aiming to surgically reduce tumor burden, followed by the localized administration of heated chemotherapeutic agents. This dual approach promises enhanced drug penetration and cytotoxicity, offering a beacon of hope for improved survival outcomes (4).

CRS + HIPEC aims to surgically remove visible tumors and eradicate microscopic disease by delivering heated chemotherapy directly into the abdominal cavity (5). Although this aggressive treatment can improve long-term survival for certain patients, it is associated with significant acute morbidity (6). Events connected to chemotherapy and surgery can be separated into two morbidity categories. Bleeding, postoperative intestinal obstruction, anastomotic leakage, wound infection, pulmonary embolism, and venous thrombosis are expected surgical consequences. Though uncommon, cytostatic agent-related morbidity in HIPEC might include thrombocytopenia, leucopenia, anemia, and liver or renal damage (6, 7). Understanding these complications is essential for enhancing patient outcomes and surgical techniques. Despite the risks, careful patient selection and the expertise of specialized institutions can lead to acceptable morbidity and mortality rates, making CRS + HIPEC a viable therapeutic option for some patients with peritoneal surface cancers (8–11).

Although CRS + HIPEC has therapeutic potential, they are associated with a wide range of complications, which can vary in severity from minor to life-threatening, pose significant clinical challenges, and affect treatment efficacy and patient recovery. This literature review aims to clarify the complications associated with CRS + HIPEC in cancer patients with PM. It examines the incidence of these complications, their underlying mechanisms, and potential strategies to reduce them. We provide a comprehensive overview to inform clinical practice and guide future research toward optimizing patient outcomes in this complex treatment landscape.

## 2 Application and indication of CRS + HIPEC

Diagnosis of PM is challenging, often resulting in underestimation of their actual incidence. While advances in diagnostic modalities like diffusion-weighted MRI have improved detection, lesions under 0.5 cm remain difficult to identify (12).

CRS + HIPEC is used to treat a variety of cancers that have spread to the peritoneum or abdominal lining. It is effective for PM, which occurs when cancer spreads from the primary tumor site to the peritoneal lining. PM is most commonly caused by colorectal cancer (CRC), appendiceal cancer, ovarian cancer, gastric cancer, and peritoneal mesothelioma (5, 13, 14). CRS + HIPEC can also treat intra-abdominal sarcomas that have spread to the peritoneum, gynecologic cancers like fallopian tube or primary peritoneal cancer, and rare cancers that are limited to the peritoneal cavity (13, 15).

The peritoneal cancer index (PCI) score is an essential diagnostic factor in determining whether cancerous patients with a PM should undergo CRS + HIPEC. Tumor burden is measured on a scale of 0 to 39. Scores ≤20 are typically considered suitable for CRS + HIPEC, though some centers may extend this to 24–26 in some instances. While the PCI score is an important prognostic tool, ongoing research aims to improve its predictive accuracy by incorporating more clinical and tumor-related variables (16, 17).

## 3 Mechanism of action of HIPEC

The combination of CRS + HIPEC is aimed at treating PM using a two-pronged approach. CRS, as the first step, entails surgically removing all visible peritoneal tumor deposits to achieve complete cytoreduction (CC-0/1 score). This debulking procedure reduces the overall tumor burden and eliminates macroscopic disease, which is often resistant to systemic chemotherapy. Achieving complete cytoreduction is critical for improved outcomes following CRS + HIPEC (18, 19).

HIPEC, as the second step, involves administering heated chemotherapy directly into the peritoneal cavity during the surgery. HIPEC works by directly applying heated chemotherapeutic chemicals to the peritoneal cavity, improving drug penetration and cytotoxicity, capitalizing on localized therapy's pharmacokinetic benefits, and reducing microscopic illness. The heated chemotherapy solution (typically at 41–43°C) is circulated throughout the abdomen for 30–90 min (18, 20, 21). Heating the chemotherapy solution improves the ability of the medicines to enter the tumor tissues. This is crucial because systemic chemotherapy frequently has limited effectiveness in treating peritoneal metastases due to inadequate blood supply and insufficient drug penetration into the peritoneal surface (19, 22).

### 3.1 Comparison of different HIPEC techniques

HIPEC techniques vary by drug delivery method and timing of administration, and each approach offers distinct advantages (23). The open HIPEC technique, the most traditional, involves leaving the abdominal cavity open for direct drug circulation, allowing the surgeon to manipulate the area for optimal drug distribution. Closed HIPEC, by contrast, seals the abdomen to enhance temperature control and minimize chemotherapy exposure for healthcare providers. Semi-closed HIPEC combines aspects of both methods, leaving a small opening to balance benefits (24, 25). Laparoscopic HIPEC is minimally invasive, suited for cases with limited disease, and promotes quicker recovery (26). Additionally, Early Postoperative Intraperitoneal Chemotherapy (EPIC), a non-heated alternative often used with HIPEC, is administered shortly after surgery over several days (27). Selecting the appropriate HIPEC technique is influenced by cancer type, disease extent, and institutional preferences, with ongoing research aimed at refining these methods and improving outcomes (28).

**Figure 1:**
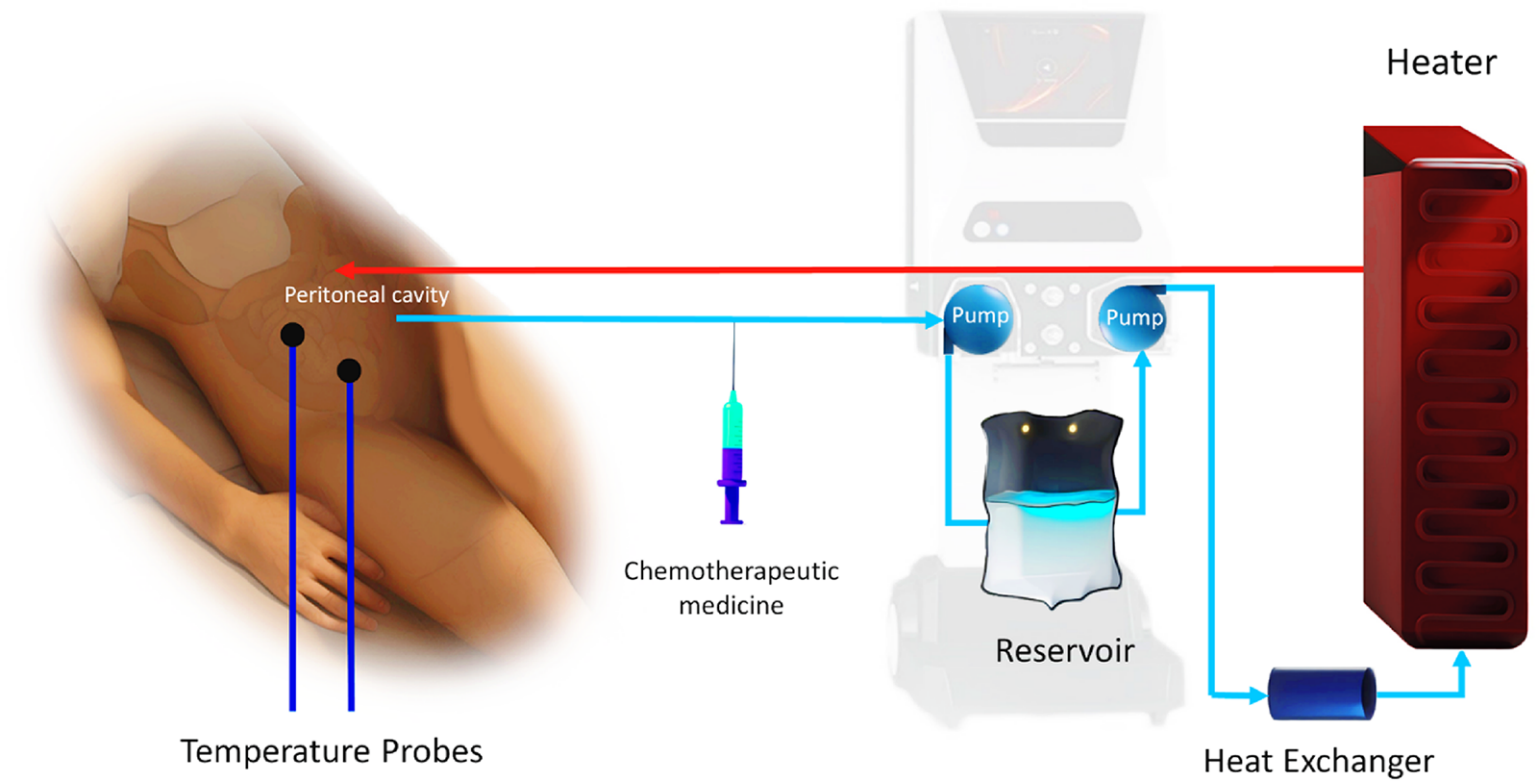
Mechanism of action of HIPEC. (Designed using CorelDRAW, 3D max, Photoshop, free sample templates, and assembled in PowerPoint).

Figure 1 illustrates a summary of the working process and mechanism of action of HIPEC.

### 3.2 Chemotherapy agents

In HIPEC procedures, various chemotherapy agents are used to treat peritoneal malignancies (23, 29). Platinum-based agents such as cisplatin, oxaliplatin, and carboplatin are commonly employed (29); cisplatin is frequently used for ovarian cancer but carries a higher risk of acute renal impairment, while oxaliplatin is preferred for colorectal cancer with peritoneal metastases due to its effectiveness in improving disease-free survival (30). Carboplatin is often chosen as a lower-toxicity alternative to cisplatin. Other agents include Mitomycin C (MMC), used for colorectal and gastric cancers with a lower risk of renal impairment, 5-fluorouracil (5-FU), sometimes used in HIPEC or EPIC, and irinotecan, which, in combination with oxaliplatin, shows promise for colorectal cancer (29). The effectiveness of these drugs in HIPEC is temperature-dependent, with optimal results typically achieved at 41°C–43°C over 60 min, especially enhancing the impact of platinum-based agents (31).

## 4 Surgical-related complications

Intraoperative complications during CRS + HIPEC are multifaceted, involving Hemodynamic instability and intraoperative metabolic changes (32–34), significant blood loss, organ injury, hyperthermia-related issues, infection risk, and technical challenges. These complications underscore the need for meticulous surgical technique, careful patient selection, and comprehensive perioperative management to optimize outcomes.

### 4.1 Technical challenges

CRS + HIPEC is known as a challenging operation among surgeons. The complexity of the procedure, including the need for extensive adhesiolysis and resection of multiple peritoneal surfaces, increases the risk of technical difficulties and prolonged operative times. The learning curve for CRS + HIPEC is steep, and outcomes are better in high-volume centers with experienced surgical teams (35). Santullo and his colleagues (36) conducted a study to examine the learning curve for CRS and the clinical outcomes of a series of patients treated by a single surgeon at a single institution. CRS's failure rate became stable after 99 instances, and complete surgical expertise was attained after 189 cases. Implementing a standardized and guided learning model is a more secure approach to expedite the learning process, decrease the occurrence of illness and death, and enhance oncologic results (36).

### 4.2 Hyperthermia-induced complications

The use of heated chemotherapy can cause thermal injury to the peritoneal surfaces and underlying tissues (37). Patients undergoing CRS + HIPEC are at risk of moderate to severe hyperthermia, which is associated with several adverse effects. A retrospective analysis of 458 patients who received CRS + HIPEC found that 32.5% had an axillary temperature ≥38°C, and 8.5% had an axillary temperature ≥39°C (hyperpyrexia) after the procedure (38). Hyperthermia can also exacerbate systemic inflammatory responses, leading to complications such as systemic inflammatory response syndrome (SIRS) (37). A study of 214 adults undergoing CRS + HIPEC found that failure to attain a temperature of 38°C at the end of chemo-perfusion or maintain it for at least 30 min was associated with worse survival, although not statistically significant (39). Cooling protocols are used during CRS + HIPEC at various institutions to reduce the risk of complications. However, these protocols are not standardized and may lead to severe hyperthermia or hypothermia. Excessive cooling could reduce the effectiveness of the chemotherapeutic agents (39). Therefore, maintaining a stable temperature during HIPEC is critical for reducing hyperthermia problems.

Intraoperative hyperthermia during HIPEC can lead to hemodynamic instability, including fluctuations in blood pressure and heart rate. The closed abdomen technique for HIPEC may offer more stable intraoperative conditions compared to the open technique, with better control of central venous pressure (CVP), pulse rate, and systolic pressure (32–34).

### 4.3 Hemorrhage and bleeding

Significant blood loss is a common intraoperative complication during CRS due to the extensive nature of the surgery. The need for blood transfusions and the risk of coagulopathy are heightened during these procedures (40–42). Marie-Elisabeth Kajdi et al. (40) reported that the median anesthesia time was 715 (range 370–1,135) minutes, and the median blood loss during the operation was 0.8 (0 to 6) liters. Significant fluid shifts required a total fluid input of 8.4 (4.2–29.4) L per patient.

### 4.4 Wound dehiscence

The incidence of wound dehiscence (WD) in patients undergoing CRS + HIPEC varies. A study reported that 3.2% of patients after CRS + HIPEC had FD, and some of those instances had additional grade III–IV problems (43). Several factors contribute to the risk of wound dehiscence in patients undergoing laparotomy (44). Older age, Component Separation Technique (CST) use, and a higher Prior Surgical Score (PSS) are independent predictors of wound complications, including wound dehiscence. Additionally, the use of doxorubicin in HIPEC has been identified as a significant predictor of fascial dehiscence (43). Risk factors for fascial dehiscence and wound complications include Doxorubicin-based HIPEC, open surgical technique, higher BMI, and component separation technique, which can lead to grade III wound complications (43). RTL-suture, a less extensive alternative to mesh reinforcement, may reduce fascial dehiscence risk without mesh-related complications, according to a study comparing different suture techniques (45). One study reported that wound dehiscence occurred in 7.1% of patients who underwent abdominal wall resection (AWR) and reconstruction as part of their CRS + HIPEC treatment, compared to 3.4% in those who did not undergo AWR (*p* = 0.028) (46). A multidisciplinary approach is crucial for managing wound dehiscence, ensuring timely treatment, enhancing patient recovery, and reducing complications in patients undergoing CRS + HIPEC (47).

### 4.5 Post-operative adhesions

Post-operative adhesions following major abdominal procedures, such as HIPEC and CRS, are common. Although the sources presented do not provide precise incidence rates for adhesions following CRS and HIPEC, it is well-established that both procedures include considerable peritoneal dissection, predisposing patients to adhesion development (48). Adhesions are a leading cause of small bowel obstruction post-surgery. In a study analyzing 730 HIPEC procedures over 15 years, bowel obstruction was identified as one of the delayed complications necessitating readmission (38). Adhesions can cause chronic abdominal pain, which significantly impacts the QoL. A study evaluating the QoL post-CRS + HIPEC found that while there was an initial decline in physical well-being, patients generally reported improvements over time (49).

The broad nature of CRS, which includes the removal of visible tumors from the peritoneal surfaces, raises the likelihood of adhesion development due to the vast regions of peritoneal damage. Heated chemotherapy can aggravate tissue inflammation and damage, promoting adhesion formation. Research on gut barrier dysfunction after CRS + HIPEC found that the surgery might cause considerable tissue damage, perhaps leading to adhesions (50). Adhesions are more common in patients who have had prior abdominal surgery because of pre-existing scar tissue and changed peritoneal morphology (38). Post-operative adhesions and deformed anatomy from prior surgery are additional complications for patients with metachronous PM treated with CRS + HIPEC (51). These have been identified as post-operative small bowel fistula risk factors (52).

### 4.6 Incisional hernia (IH)

The incidence rates of IH after CRS + HIPEC vary across studies but generally fall between 7% and 17%. For instance, one study reported an IH incidence of 17% in a cohort of 155 patients, with a median time to diagnosis of 245 days post-surgery (53). Another study found an IH incidence of 7.8% in patients undergoing CRS + HIPEC, which is comparable to the rates observed after other major abdominal surgeries (45). After CRS + HIPEC, IH significantly impacts health-related QoL, causing discomfort, pain, and physical activity limitations, affecting overall well-being (54). Incisional hernia (IH) is associated with risk factors for both surgery and the patient. Significant independent factors include female gender and age, with patients 50–64 years old and older showing increased hazard ratios for developing IH (53). A history of prior abdominal operations and a higher body mass index (BMI > 30) both increase the risk because of the accumulated stress and possible weakening of the abdominal wall (45). The incidence of infection handled surgically has a substantial influence; research demonstrating a 2% IH incidence in the RTL group compared to 13% in the regular closure group suggests that employing a reinforced tension line (RTL) suture method can lower IH rates (55). Furthermore, decreased incidence of infection (IH) has been linked to using a 4:1 suture length to wound length (SL) ratio during fascial closure. Research that used this ratio found that IH incidence was 13.0% in patients closed with it, compared to 34.9% in those who used traditional closure techniques (56). Regular follow-up and monitoring are crucial for the early detection and management of IH, with cross-sectional imaging and physical examinations essential for postoperative identification (45).

### 4.7 Surgical-induced infection

Surgical Site Infections (SSI) are common occurrences after CRS + HIPEC procedures. According to a study, surgical site infections (SSIs) were observed in 35.7% of patients, making it the most prevalent type of infection. Central Line-Associated Bloodstream Infections (CLABSI) were the second most predominant, affecting 26.2% of patients (57). A study found a substantial occurrence of Abdominopelvic infections following HIPEC, with an incidence rate of 5.2%. 4.8% of patients exhibited pulmonary infections (3). Intraoperative bacterial contamination is a concern, and systematic bacterial sampling during CRS + HIPEC can help predict postoperative infectious complications (58).

Several factors, including preoperative conditions (51), nutritional status (57), blood loss, and ascites volume, increase the risk of postoperative infections in CRS + HIPEC patients. Malnourished individuals have a higher rate of infectious complications compared to well-nourished patients. Blood loss of 350 ml or more during surgery and ascites volume of ≥300 ml are linked to an increased risk of infection. Anemia, obesity, and a history of intravenous chemotherapy toxicity also contribute to more excellent infection rates (3, 51, 57).

The microbial spectrum of infections post-CRS + HIPEC includes a variety of fungi, gram-positive and gram-negative bacteria (59). A study reported that *Staphylococcus epidermidis* was the most common gram-positive bacterium isolated. Vancomycin was the most effective antibiotic against gram-positive bacteria, with a sensitivity of 98.4%. Levofloxacin was the most effective antibiotic for gram-negative bacteria, with a sensitivity of 68.5%. Fluconazole was the most effective antifungal agent, with a sensitivity of 83.3% (3).

## 5 Anesthetic-related complications

CRS + HIPEC presents major anesthetic problems, including hemodynamic instability, respiratory complications, renal failure, coagulation abnormalities, and thermal management issues. Effective perioperative management necessitates enhanced monitoring, meticulous hydration and temperature management, and a multidisciplinary approach to address problems and improve patient outcomes.

### 5.1 Hemodynamic instability

Hemodynamic instability during CRS + HIPEC operations occurs due to considerable blood loss and fluid shifts, necessitating careful fluid management and transfusions. The HIPEC phase exacerbates these challenges by increasing intra-abdominal pressure and lowering venous return and cardiac output. In addition, the heated chemotherapeutic solution causes vasodilation, which reduces systemic vascular resistance and mean arterial pressure while increasing heart rate and cardiac output (60, 61).

### 5.2 Intraoperative metabolic changes

HIPEC injection following cytoreductive intraperitoneal cancer surgery induces considerable alterations in internal homeostasis, particularly in the patient's temperature, blood glucose, and lactic acid (33). Dyselectrolytemia and lactic acidosis are possible consequences of the treatment, as chemotherapeutic drugs and physiological stress can produce substantial electrolyte imbalances and metabolic acidosis, requiring close monitoring and correction (61, 62).

### 5.3 Respiratory complications

Respiratory complications during HIPEC include cephalad diaphragm displacement caused by increased intraabdominal pressure, which compromises respiratory mechanics and needs protective mechanical ventilation techniques (60, 61). Furthermore, HIPEC-induced hyperthermia increases systemic oxygen demand, resulting in more excellent metabolic activity and higher end-tidal CO2 levels (62).

### 5.4 Coagulopathies complications

Coagulopathies are rather prevalent with CRS + HIPEC. HIPEC-induced hyperthermia can cause coagulopathies, increasing the risk of hemorrhagic complications. Coagulation parameters must be carefully monitored and managed during the operation (40, 62). A comprehensive review and meta-analysis revealed a minimal incidence of postoperative bleeding within 30 days and VTE within 90 days with CRS + HIPEC for colorectal cancer with PM (63). Coagulopathies were among the complications found in another study of 1,321 sequential CRS + HIPEC operations, stressing the importance of cautious perioperative treatment (64).

**Figure 2:**
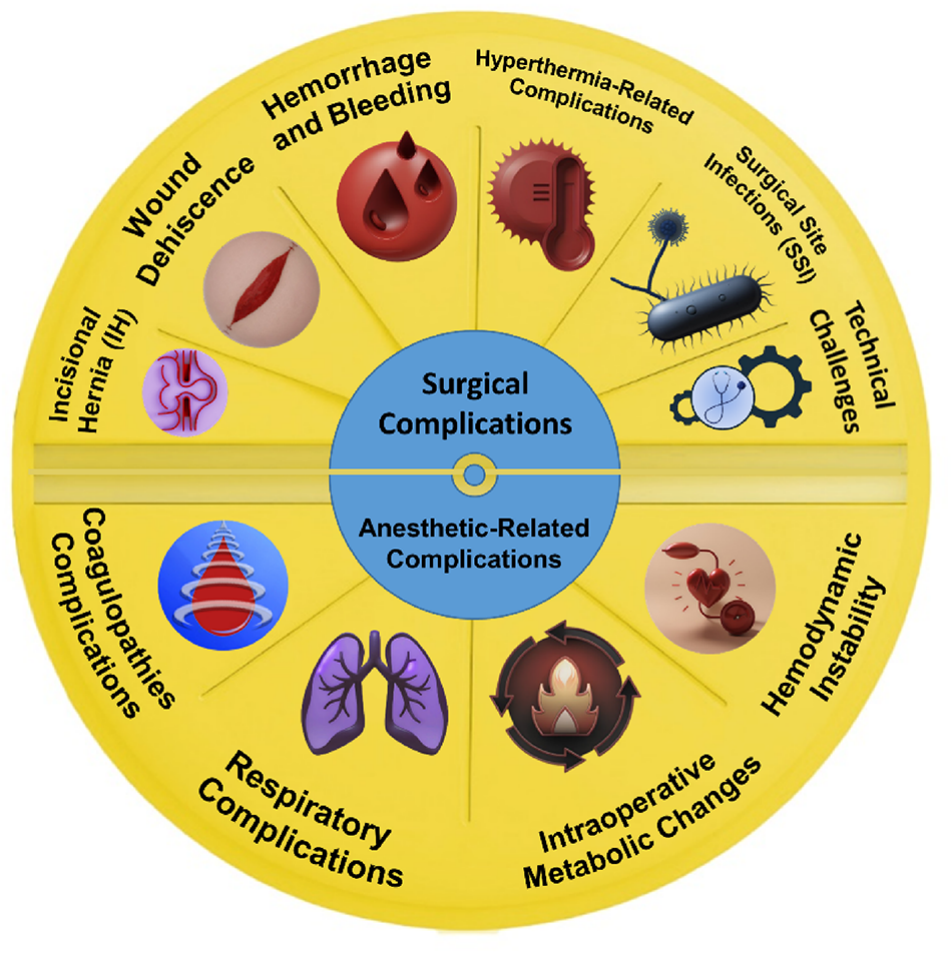
Summary of surgical and anesthetic-related complications. (Designed using CorelDRAW, 3D max, Photoshop, free sample templates, and assembled in PowerPoint).

Figure 2 summarizes intraoperative complications, including surgical and anesthetic complications, that follow CRS + HIPC (Figure 2).

## 6 Chemotherapy-related and systemic complications

### 6.1 Hematologic toxicity and complications

Hematologic toxicity, such as neutropenia and thrombocytopenia, is a well-recognized complication of HIPEC. A study reported that the incidence of post-procedure thrombocytopenia was 46%, with severe (grade 3–4) thrombocytopenia occurring in 4% of patients (65). Another study found that mitomycin C was an independent risk factor for severe hematologic toxicity, including medullary toxicity, neutropenia, and thrombocytopenia (57). This study of 96 patients undergoing CRS + HIPEC found that 77.1% experienced hematological complications, including leukopenia (8.3%), anemia (66.7%), and coagulopathy (22.9%). Complications were more common in ovarian cancer patients and those treated with doxorubicin or cisplatin, leading to longer ICU stays for some. Bleeding issues were minor and managed conservatively. Despite these complications, the median ICU stay was five days, and the mortality rate was only 1%, with most complications resolving without impacting overall mortality or hospital stay (66).

Elderly patients over 60 and those with a history of chemotherapy are particularly at risk and should be closely monitored post-CRS + HIPEC (67). Neutropenia, primarily associated with using mitomycin C (MMC), is a severe complication that increases susceptibility to infections and sepsis. Risk factors for neutropenia include anemia, obesity, previous toxicity to intravenous chemotherapy, and female sex (51). Myelosuppression is another significant side effect of MMC, occurring in about 28% of cases, and severe instances can lead to life-threatening infections (68).

### 6.2 Systemic infections and sepsis

Following CRSH + IPEC procedures, sepsis and infection emerge as the primary contributors to mortality associated with treatment (69). Due to the immunosuppressive effects of chemotherapy and the extensive nature of the surgery, patients are at increased risk of developing systemic infections and sepsis (34, 70). To enhance diagnostic precision, Procalcitonin (PCT) was implemented at an earlier stage to identify postoperative infections. The diagnostic efficacy of serum parameters during the initial postoperative period is constrained and significantly influenced by the extent and nature of the surgical procedure (71). Toward the end of the first postoperative week, markers can help manage patients during peak surgery-related inflammation. Procalcitonin is made by C cells in the thyroid gland and other cell types when there is a bacterial infection. PCT is triggered by bacterial endotoxins and lipopolysaccharides and indirectly by inflammatory markers like tumor necrosis factor-alpha and interleukin-6. It is peculiar in diagnosing bacterial infections and sepsis (72).

The microbial spectrum and antibiotic sensitivity results help clinicians manage postoperative infections in PMP patients (3). Timely identifying adverse events is crucial in reducing failure to rescue after CRS + HIPEC. Accurate detection of infectious complications post-CRS + HIPEC is critical. A patient's clinical presentation remains vital in surgical assessment, while hematological parameters can assist in screening or specifying complications (71).

A study of 127 individuals with CRS + HIPEC found that 41.7% had infective complications (ICs) and 12.6% had non-infective complications (NICs). Infective consequences were substantially related to higher C-reactive protein (CRP) levels after surgery, particularly between postoperative days 7 and 10 (73). Yang et al. studied 482 pseudomyxoma peritonei (PMP) patients, finding 17.0% infected after CRS + HIPEC. The most common infections were central venous catheter (CVC) (8.1%) and abdominal-pelvic (5.2%). 29 microbes were isolated, including Staphylococcus epidermidis, Gram-positive and Gram-negative bacteria, and funguses. Antibiotic sensitivity results indicated vancomycin for Gram-positive bacteria (98.4%), levofoxacin for Gram-negative bacteria (68.5%), and fuconazole for fungus (83.3%). Risk factors for infection included blood loss ≥350 ml (*P* = 0.019) and ascites volume ≥300 ml (*P* = 0.008).

The risk for increased postoperative complications has been closely examined when bevacizumab, a targeted therapeutic, is used in conjunction with systemic chemotherapy before CRS + HIPEC. Preoperative bevacizumab did not substantially raise the risk of severe morbidity or death, according to one research, indicating that it is safe for use in neoadjuvant situations. On the other hand, different study found that patients who got bevacizumab had a greater risk of grade 3–5 problems than those who did not (74).

### 6.3 Nephrotoxicity

The chemotherapeutic agents used in HIPEC, such as high-dose cisplatin, can cause nephrotoxicity, leading to renal impairment. Additionally, fluid management strategies, particularly hydroxyethyl starch colloid solutions, may have negatively impacted renal function (40, 61). Acute kidney injury (AKI) is a common complication following HIPEC + CRS, whereas post-HIPEC chronic kidney disease (CKD) is rare and less investigated. HIPEC was identified as a high-risk factor for postoperative AKI because of the direct effects of nephrotoxic chemotherapeutic medicines, fluid distribution, splanchnic vasodilation generated by hyperthermia, and arterial hypotension (75). The incidence of AKI in Some studies shows a rate as high as 20%, especially when cisplatin is administered (76–78). AKI following HIPEC accounts for 1%–48% of cases and is linked to 50% of serious complications (75, 79). Following CRS + HIPEC, AKI is linked to a greater incidence of severe complications, which can lead to more extended hospital stays and higher death rates (78, 80). For a long time, AKI was believed to be a reversible condition. However, it did raise the risk of death and the development of CKD, which came after end-stage renal disease (ESRD) (81). AKI is thought to be a frequent side effect following CRS + HIPEC.

In a retrospective clinical evaluation of 153 HIPEC patients, 31.8% experienced AKI. HIPEC regimens using cisplatin were a significant risk factor for AKI (*p* < 0.001). Angiotensin receptor blocker use raised preoperative creatinine, and increased preoperative urea levels were also independent risk factors (82). Chemotherapeutic agents can cause significant metabolic disturbances, including hypocalcemia and hyperglycemia, which require careful monitoring and management (70). A study identified intraoperative use of parecoxib during cisplatin-based HIPEC as a significant risk factor for postoperative AKI and CKD, with 30.9% of patients developing CKD. The findings highlight the importance of recognizing and avoiding specific risk factors to improve long-term renal outcomes for patients undergoing cisplatin-based HIPEC (75).

### 6.4 Chemotherapy-related GI complications

Gastrointestinal complications from HIPEC + CRS include digestive fistulas and perforations, as the combination of hyperthermia and high-dose chemotherapy can impair healing, increasing the risk of anastomotic leaks and small bowel perforations, leading to severe morbidity. Enterocutaneous fistulas are another serious postoperative complication, significantly impacting the patient's QoL and necessitating prolonged medical care (51, 70, 83).

### 6.5 Hepatotoxicity

Chemotherapy-induced hepatotoxicity is a significant problem in oncology, especially for patients undergoing intense therapies like CRS + HIPEC. A study of 301 patients indicated that 57.71% of patients in the CRS + HIPEC group developed hepatotoxicity, compared to 42% in the surgery-alone group. The addition of HIPEC resulted in a considerable rise in liver-related problems (84). Several factors contribute to the risk of hepatotoxicity in CRS + HIPEC. The type of chemotherapeutic agents used, particularly the combination of cisplatin and docetaxel (Cis + Doc), is a primary risk factor. Additionally, open HIPEC techniques and procedures lasting longer than 60 min are associated with a higher incidence of hepatotoxicity. Post-surgical gastrointestinal complications also correlate with increased hepatotoxicity (84).

## 7 Cardiovascular (CV) complications

### 7.1 Myocardial infarction (MI)

Elevated brain natriuretic peptide (BNP) levels have been discovered as an early indicator of individuals at risk of having cardiac problems following CRS + HIPEC. High BNP levels are associated with poor cardiac outcomes in the postoperative phase (85). Hemodynamic instability, substantial blood and fluid loss, metabolic derangements, and prolonged surgical duration can all lead to cardiac events, including cardiac arrest, right after surgery. Furthermore, rebound hypothermia and electrolyte abnormalities, notably hypokalemia, after the HIPEC phase might heighten the risk of cardiac problems (86). Postoperative troponin elevation, particularly cardiac troponin I (cTnI) levels more than 2–3 times the upper limit of normal, is associated with an increased risk of significant circulatory complications (87).

### 7.2 Cardiomyopathy

Stress-induced cardiomyopathy following CRS + HIPEC is a rare complication that might arise following significant operations such as CRS + HIPEC (88).

A case report details the development of stress-induced cardiomyopathy, also known as Takotsubo cardiomyopathy, in a 43-year-old woman following CRS + HIPEC surgery for pseudomyxoma peritonei. This rare but notable complication presented with acute chest pain, hemodynamic instability, electrocardiographic changes, and severely reduced left ventricular systolic function, closely resembling acute coronary syndrome despite normal coronary arteries. The precise mechanism remains unclear, but it is believed to be linked to the significant surgical stress and inflammatory response associated with the CRS + HIPEC procedure (88).

### 7.3 Deep vein thrombosis (DVT)

In a study of 250 patients who underwent CRS for ovarian cancer, with 124 also receiving HIPEC, 20 patients (8%) developed deep vein thrombosis (DVT) within 30 days post-surgery, and an additional 3 patients (1.2%) were diagnosed with DVT after 30 days. This makes DVT the most common significant postoperative complication in this group. Several risk factors contribute to this high incidence, including the anatomical confinement of vessels and lymphatics in the pelvic region and restricted movement during surgical dissection. The extensive surgical trauma to pelvic vessels and lymphatics during CRS + HIPEC is a significant factor in the development of DVT, highlighting the need for vigilant monitoring and preventive measures in these patients (89).

The systematic review and meta-analysis by Mogensen et al. found that the overall 30-day incidence of postoperative bleeding after CRS + HIPEC for PM from colorectal cancer was 4.2% [95% CI 2.6%–6.2%]. The 90-day incidence of venous thromboembolism (VTE) was 2.7% [95% CI 1.0%–5.2%]. They concluded a low risk for both postoperative bleeding within 30 days and VTE within 90 days following CRS + HIPEC for PM from colorectal cancer (63). A systematic physiotherapy program, including active exercises and intermittent pneumatic compression, has been proven safe and feasible in preventing VTE in patients undergoing CRS + HIPEC, significantly reducing clinically symptomatic VTE events (63).

### 7.4 Pulmonary embolism (PE)

Pulmonary Embolism (PE), while less common than DVT, is still a notable complication. Several risk factors have been found for the development of PE after CRS + HIPEC, including preoperative pleural effusion, completeness of cytoreduction (CC) score of 0, increased amount of blood transfusions, prolonged surgical length, cancer diagnosis, and advanced stage (90, 91). In a study analyzing 730 HIPEC procedures over 15 years, PE was identified as one of the delayed complications necessitating readmission, alongside bowel obstruction and rectovaginal fistulas (92). In a smaller cohort of 20 patients undergoing CRS + HIPEC with coloanal/ileoanal anastomosis, one patient died of a pulmonary embolism on postoperative day 7, highlighting the potential lethality of this complication (93).

To conclude, patients receiving CRS + HIPEC are at a high risk of CV complications due to the extent of the operation and the resulting physiological alterations. To lower the risk of cardiac events in this high-risk patient population, comprehensive preoperative evaluations, vigilant monitoring of hemodynamics throughout surgery, and adherence to standardized fluid management procedures are essential.

## 8 Gastrointestinal complications

For patients undergoing CRS + HIPEC, particularly those with metastatic colorectal cancer, gastrointestinal complications pose a severe risk, significantly impacting outcomes with issues such as enterocutaneous fistulas, intestinal blockages, anastomotic leaks, and digestive fistulas. Effective management necessitates preoperative optimization, precise surgical techniques, close postoperative monitoring, and a multidisciplinary care approach (94–96). Several risk factors contribute to these gastrointestinal issues: the extensive nature of CRS, which involves resecting multiple organs and performing complex procedures, increases the risk of complications. Additionally, chemotherapeutic agents used in HIPEC, like oxaliplatin and mitomycin C (MMC), can impair healing and elevate the risk of anastomotic leaks and perforations. Patient-related factors, including prior abdominal surgeries, comorbidities, and nutritional status, are also crucial in determining the likelihood of postoperative gastrointestinal problems (94, 95).

### 8.1 Fistulas and perforations

Digestive fistulas, particularly enterocutaneous fistulas, are well-known complications of CRS + HIPEC. The prevalence of these fistulas differs among research studies. One study, for example, found an 8.6% incidence of peritoneal carcinomatosis in 184 individuals treated with CRS + HIPEC (96). Another study found that the most prevalent and clinically critical gastrointestinal problems are small bowel perforations and anastomotic leaks, with a reported rate of grade III/IV GI events ranging from 4.5% to 19% (51). Small intestine perforations are a severe complication caused by a variety of factors, including partial-thickness mechanical damage to intestinal surfaces, focal heat injury at the tip of the inflow catheters, the suctioning effect of the outflow catheter, and postoperative shrinkage of infiltrating metastatic nodules on the intestinal wall due to HIPEC's antiblastic effect (51, 83).

Several risk factors have been identified for the development of fistulas and perforations, including a high PCI, which indicates extensive disease; previous surgeries that resulted in adhesions and distorted anatomy; and the use of hyperthermia and high-concentration chemotherapy, which can disrupt physiological healing processes (83, 97). In one trial, 87.5% of patients experienced spontaneous fistula closure, which lasted an average of 18 days (96). The mortality rate from postoperative peritonitis caused by intestinal fistulas can be substantial, ranging from 10% to 47%, but it can be lowered with immediate surgical treatment and a multidisciplinary approach (98).

### 8.2 Anastomotic leaks

Anastomotic leaks are one of the most severe gastrointestinal consequences after CRS + HIPEC, with reported incidences ranging from 5.2% to 10.3%. Small intestinal anastomoses are the most prevalent source of leaks, accounting for roughly 44% of cases (83, 99). These leaks can cause severe complications such as peritonitis, sepsis, and the need for reoperation, which can have a considerable impact on the patient's rehabilitation and overall prognosis (94, 95). There are several known risk factors for anastomotic leakage following HIPEC (83, 100). Patient-related factors such as older age at surgery (OR 1.40), smoking, comorbidities (assessed by the Charlson Comorbidity Index), and preoperative nutritional status (100). Disease-related factors include having a pelvic peritonectomy (OR 2.33) and having a higher PCI (OR 1.04 per unit increase). Several treatment-related factors include the use of bevacizumab within 60 days of surgery (OR 6.13), the use of higher doses of cisplatin (>240 mg) during HIPEC (OR 3.53), the presence of stapled anastomoses (OR 2.59), a higher number of anastomoses, and colonic resection. Furthermore, receiving red blood cell transfusions during surgery increases the risk of gastrointestinal perforations (83, 100, 101).

### 8.3 Intestine obstruction

Small intestine obstruction is frequently followed by CRS + HIPEC, with incidence rates differing among studies. For instance, one study revealed that 19.9% of patients were re-admitted due to SBO, attributing 57.5% of cases to adhesions and 42.5% to malignancies (102). Another study observed that the cumulative incidence of SBO readmission was 24% at one year and increased to 38% at two and three years (103).

Postoperative ileus (POI), temporary paralysis of the bowel, is a common complication after CRS + HIPEC and can delay recovery. The incidence of POI ranges from 15% to 54%, accounting for approximately 15% of early hospital readmissions (104).

Risk factors contributing to bowel obstruction after CRS + HIPEC include older age and existing health conditions, which elevate the risk. Nutritional status and previous abdominal surgeries also influence susceptibility. Disease-related factors such as higher PCI scores and specific malignancies like colorectal cancer increase the likelihood of developing obstructions (102). Treatment factors include the use of intraperitoneal chemotherapy agents such as mitomycin C, which significantly heightens the risk of obstruction. Surgical factors, including the extent of resections and the number of anastomoses performed, are crucial predictors of complications (8, 102, 105). Intraoperatively, increased gastrointestinal wall thickness due to tissue trauma and swelling during CRS + HIPEC leads to longer operative times and hospital stays, thereby contributing to the onset of postoperative ileus (104).

Conservative management of SBO includes bowel rest, nasogastric decompression, and intravenous fluids. This approach resolved obstruction in 76.7% of patients either spontaneously or through surgical intervention (102). Surgical intervention is required in a subset of patients with SBO. In one study, 28.7% of patients with SBO required surgery (102). The need for re-operation due to bowel obstruction or other complications was reported in 9% of patients in a large series (8). Bowel obstruction resolution is crucial for patient outcomes, but high-grade complications, including bowel obstructions, have an adverse prognostic factor for survival (8). Preventive measures involve careful surgical technique to minimize adhesions, judicious use of chemotherapy agents, and close postoperative monitoring to detect and manage complications early (94, 95).

## 9 Respiratory (pulmonary) complications

CRS + HIPEC may increase the risk of respiratory problems, such as ARDS, pneumonia, pleural effusions, respiratory distress, and diaphragmatic dysfunction (106–108). Pleural effusions can develop as a result of significant surgical dissection, particularly when diaphragmatic peritonectomy is undertaken. Fluid collection in the pleural space can impede respiratory function, necessitating drainage (106). Due to the more extended surgical recovery period, immunosuppression from chemotherapy, and possible aspiration events, patients receiving CRS + HIPEC are more likely to develop pneumonia. Pneumonia can impede breathing even more and take longer to heal (106). In these patients, Respiratory distress, characterized by shortness of breath and hypoxemia, can occur due to various factors, including pleural effusions, pneumonia, and the physiological stress of the procedure (106). Diaphragmatic dysfunction and poor respiratory mechanics can result from diaphragmatic resection, also known as peritonectomy, which is frequently required during CRS (107, 108) Studies have demonstrated that, in skilled surgeons, diaphragmatic excision does not always entail a higher risk of respiratory problems.

Pulmonary complications after CRS + HIPEC are comparatively frequent, with reported incidences varying throughout research. One retrospective study reported that 17% of patients (72 out of 417) developed severe postoperative pulmonary complications, defined as Grade ≥3 according to the Clavien–Dindo classification system (109). Another retrospective study found that pulmonary complications, such as pneumonia and respiratory failure, are common in the period after abdominal surgery, though the exact incidence was not specified (106). A retrospective study from the University Hospital of Arrixaca identified that only 3.2% of patients (8 out of 247) developed postoperative respiratory complications after undergoing CRS + HIPEC for peritoneal carcinomatosis (110). A high PCI score (>14) and diaphragmatic peritonectomy are independent risk factors for respiratory problems (110). Significant risk variables included diaphragmatic resection and full-thickness diaphragmatic damage, with an odds ratio of 5.393 (95% CI: 2.924–9.948, *p* < 0.001) (109). The Uppsala University Hospital study found that severe postoperative pulmonary complications, in combination with non-pulmonary complications, contributed to decreased overall survival (HR 2.285, 95% CI: 1.232–4.241, *p* = 0.009) (109). Effective management options to mitigate and resolve these problems include preoperative optimization, cautious intraoperative management, postoperative monitoring and support, and a multidisciplinary approach.

## 10 Efficacy of CRS + HIPEC in pediatric patients

HIPEC is usually tolerated in pediatric patients, with no documented postoperative death and just brief postoperative problems. However, because there have been few clinical trials and small sample sizes, it is still unclear how this may affect overall survival in the long run (111). In pediatric patients with malignant peritoneal mesothelioma, complications included acute kidney injury, hyperbilirubinemia, bilateral pleural effusions, and pneumothorax requiring chest tube placement. Despite these complications, many patients had favorable long-term outcomes (104). A study investigated the influence of the extent of cytoreduction on pediatric and adolescent patients who underwent CRS + HIPEC. The results indicate that attaining full cytoreduction is essential for enhancing outcomes in these individuals. The study emphasizes the significance of precise surgical approaches in eliminating visible tumors since this greatly influences the effectiveness of the treatment (112).

## 11 Morbidity and mortality, overall safety and outcomes

Long-term follow-up studies have revealed that, while CRS + HIPEC can increase survival rates, they are also linked to considerable morbidity. For example, one research found that CRS with systemic chemotherapy resulted in a median survival of 32.4 months, with a high frequency of comorbidities (113).

In significant studies of diverse cancer types, overall morbidity rates for grade III-IV complications range from 22%–34% (68). Morbidity rates can be higher for specific cancer types, such as gastric cancer (around 40%), and lower for ovarian cancer (around 12%–15% for severe morbidity) (68). Factors associated with increased morbidity include advanced age, hypoalbuminemia, poor performance status, obesity, higher PCI, incomplete cytoreduction, and the need for multiple visceral resections or anastomoses.

In large series, mortality rates typically range from 0.8 to 4.1%, with more excellent rates recorded for gastric cancer (about 5%) and lower rates for ovarian cancer (0.8%–2.5%) (9, 68). Respiratory complications and bleeding are the most common causes of mortality (8). Significant predictors of mortality include advanced age, gastric cancer histology, higher PCI, incomplete cytoreduction, and the need for multiple visceral resections (8, 9).

CRS + HIPEC can be performed safely and with acceptable morbidity and death rates with proper patient selection and experience in high-volume institutions (8). Comparative analysis of national datasets has indicated that CRS + HIPEC has lower morbidity and mortality rates than other major oncologic surgeries such as Whipple, hepatectomy, and esophagectomy (10). Having a learning curve of at least 110–150 instances is critical for improving outcomes and lowering problems (11). Patient selection, which includes characteristics such as PCI, cytoreduction completeness, and performance status, is critical in predicting surgical results (10, 11).

While HIPEC has shown promise in improving survival rates for patients with peritoneal surface malignancies, it is a costly procedure, leading to ongoing investigations into its cost-effectiveness. Key factors influencing HIPEC's economic viability include recurrence-free survival, length of hospital stay, healthcare system structure, and institutional experience. Recurrence-free survival strongly impacts incremental cost-effectiveness, and longer hospital stays increase overall costs. Additionally, variations health insurance and procedural efficiency that improves with hospital experience further shape HIPEC's financial feasibility. Despite the high resource demands, studies indicate that HIPEC, when combined with cytoreductive surgery, can be cost-effective (114–117).

## 12 Impact on survival and quality-of-life (QoL)

The occurrence of high-grade postoperative complications (grade 3–4) has been shown to impact overall survival in patients with PM from CRC negatively. For instance, a study reported that high-grade complications were associated with worse overall survival (HR, 1.86, 95% CI, 1.22–3.51; *P* = 0.044) (118). Additionally, these complications can delay or preclude the administration of adjuvant chemotherapy, further affecting long-term outcomes (118, 119).

Despite the high morbidity associated with CRS + HIPEC, including wound complications, the procedure is associated with improved survival rates in selected patients. For instance, the 5-year survival rate for patients with PM from colorectal cancer undergoing CRS + HIPEC can exceed 45% (120). However, the high rate of complications, including wound dehiscence, underscores the need for careful patient selection and management (120).

## 13 Conclusion

Table 1 illustrates a summary of recent studies that report complications following CRS + HIPEC.

**Table 1:** Complications and consequences following CRS + HIPEC.

Author/year	Baseline characteristics	Primary tumor	PCI score (median)	Outcome	Complication and consequence	Conclusion
Cardi et al. (57)	- Population: 200 - Age: 61.3 (32–75) - Male: 43 - BMI: 18.5–24.99	- Ovarian: 101 (50.5%) - Colorectal: 52 (26%) - Gastric: 16 (8%) - Pseudomyxoma peritonei: 11 (5.5%) - Malignant mesothelioma: 9 (4.5%) - Breast: 6 (3%) - Endometrial: 5 (2.5%)	16.5 (6–29)	Correlation between malnourished patients who undergo HIPEC + CRS with postoperative infectious complications	- HIPEC-related: 9 Renal (6), Hematological (3) - Infectious complications: Infections (42), Sepsis (2), Abdominal abscess (8), Bloodstream infections: (11), Wound infection (15), Pneumonia (6), - Noninfectious complications: 37 Acute postoperative pancreatitis (4), Pleural effusion (10), DVT (2), TIA (1), PE (2), Respiratory failure (4), Bowel perforation (4), Anastomotic dehiscence (1), Colostomy necrosis (1), Urinary fistula (2), Peritoneal bleeding (2), Gastric bleeding (1), Acute MI (3) - Uneventful recovery: 112 (56%) - Mortality rate: 3.5%	Malnourished patients are more prone to postoperative infectious complications.
Yang et al. (3)	- Population: 482 - Age: 55 (25–75) - Male: 211 (43.8) - BMI: 23 (15.2–40)	Pseudomyxoma peritonei	29 (1–39)	- Post-operative - Identify common infections - Identification of the most effective treatment using antibiotic sensitivity test - Identification of independent risk factors for postoperative infection	- CVC infection (39), Abdominal and pelvic infection (25), Pulmonary infection (23), Surgical wound infection (10), Urinary system infection (5), Blood culture bacteria/fungi positive (unknown infection site) (5), ≥2 Infection sites (21) - 29 kinds of microbes isolated from the culture: 13 kinds of Gram-positive bacteria (13), kinds of Gram-negative bacteria (12), kinds of funguses (4) - Median OS: 79.3 m - Risk factors for postoperative infection: ascites volume ≥300 ml and intraoperative blood loss volume ≥350 ml - Mortality: 173 (35.9%)	Pseudomyxoma peritonei patients may have an increased risk of infection.
Kang et al. (38)	- Population: 458 - Age: 57.1 (±11.5) - Male: 176 (38.43%) - BMI: 24.16 (±3.66)	Appendix tumor: 9 (1.96) Gastric cancer: 196 (42.79) Colorectal cancer: 55 (12.01) Pancreatic cancer: 2 (0.44) Gynecological malignancies: 183 (39.96) Peritoneal malignant tumor/mesothelioma: 13 (2.84)	NR	Determining the risk factors of increasing the temperature	- Axillary temperature of not below 38°C: 149 (32.5) - Axillary temperature of not lower than 39°C: 39 (8.5) - Female gender, gynecological malignancies, type of chemotherapy drug, increased postoperative neutrophil percentage, and a sharp drop in postoperative pre-albumin was associated with the incidence of a temperature increase and axillary temperatures of >38°C	The influence of different types of chemotherapy drugs is an independent risk factor for a temperature increase following CRS-HIPEC.
Wenzelberg1 et al. (45)	- Population: 129 - Age: 57 (26.8) - Male: 62 (48%) - BMI: 26.0 (4.1)	- Colon cancer: 64 - Appendix cancer: 33 - Rectal cancer: 15 - Peritoneal pseudomyxoma: 12 - Small bowel cancer: 3 - Fallopian tube cancer: 1 - Malignant mesothelioma: 1	11 (8.1)	- Evaluation of the incisional hernia incidence 12 ± 3 months after surgery - Compare the IH incidences between the two closure techniques		- An incisional hernia incidence is not higher than laparotomies - Suggesting an advantage with reinforced tension line suture.
Campos et al. (110)	- Population: 247 - Age: 58 (27–79) - Male: 16 (6.7%) - BMI: NR	Ovarian carcinoma or platinum-sensitive recurrences, colorectal cancer, gastric cancer, non-ovarian gynecological tumors, peritoneal sarcomatosis, peritoneal pseudomyxoma, malignant peritoneal mesothelioma	11	Respiratory complications According to the NCI-CTCAE	- Respiratory complication according to NCI-CTCAE: 8 (3.2%) I. Grade II: 1 II. Grade III: 6 III. Grade IV: 1 - Pleural effusions: 72 - Symptomatic pleural effusion according to NCI-CTCAE: 6 I. Grade III: 5 II. Grade IV: 1	Patients requiring diaphragmatic peritonectomy as part of their CRS almost universally develop post-operative pleural effusion less than 10% of these require pleural drainage to alleviate their respiratory symptoms.
Merboth et al. (121)	- Population: 15 - Age: 59.4 (50.1_69.3) - Male: 7 (46.7) - BMI: 23.3 (21.9–26)	Advanced gastric cancer	7	- Surgical complications - Rate of reoperation, - Rate of mortality - Total recurrence rate - Rate of peritoneal recurrences - Median survival - 1year survival - 2 year survival - OS time after surgery - Median OS time from date of diagnosis	- Anastomotic leakage: 1 (6.7) - Duodenal stupm insufficiency: 0 - Intra-abdominal abscess: 2 (13.3) - Wound infection: 3 (20) - Bleeding: 1 (6.7) - Bacteremia/Sepsis: 1 (6.7) - Thromboembolic events: 1 (6.7) - Respiratory insufficiency: 2 (13.3) - Length of stay (day): 15 (13–20) - Reoperation: 2 (13.3) - Readmission: 1 (7.1) - 30-day mortality: 1 (6.7) - Total recurrence rate: 85.7% - Rate of peritoneal recurrences: 57.1 - 1-year survival: 60.0% - 2-year survival: 26.7% - OS time after surgery: 12 months (9–26) - Median OS time from date of diagnosis: 21 months	This technique did not lead to increased complications and postoperative mortality.
Lu et al. (122)	- Population: 158 - Age: 60 (±11.9) - Male: 63 (±39.9) - BMI: 22.7 (±3.2)	- Colorectal cancer: 32.3% - Gastric: 26.6 - Appendiceal: 20.9% - Ovarian: 12.7% - Other cancers: 7.6%	17 ± 7.8	- Identify risk factors for AKI - Investigate the prevalence of AKI-to-AKD transition - Identify possible factors impact short-term outcomes - Identify possible factors impact long-term outcomes	- Postoperative AKI: 34 (21.5%) stage I. stage I: 20 (13.3) stage II. stage II: 6 (3.8) stage III. stage III: 7 (4.4) - AKI patients coincided with the AKD diagnosis: 20 (61.8) - During the 90-day follow-up for the AKD group, 42.8% of patients were diagnosed with CKD - 30-Day mortality: 5 (3.2) - ICU length of stay (days): 1.1 ± 2.7 - Length of stay (days): 19.4 ± 11.1 - 30-day mortality: 5 (3.2%)	Incidence of AKI in patients is high, and the transition to AKD is a common outcome following AKI, which confirms the association with more risk for both in-hospital mortality consulting from renal function failure and CKD progression.
Spiegelberg et al. (123)	- Population: 102 - Age: 57.2 (23–80) - Male: 60 (59%) - BMI: 25.3 (15.9–39.6)	Colorectal cancer	- Oxaliplatin group: 8 (0–30) - Mitomycin-C group: 12 (0–39)	Evaluation of therapeutic benefits, complications, and prognostic factors	- Rate of complications: 57 (56) - Pneumonia: 5 (5) - Re-intubation: 2 (2) - Pulmonary embolism/thrombosis: 2 (2) - Hematoma: 2 (2) - Postoperative hemorrhage 4 (4) - Intestinal atony: 23 (23) - Wound infection: 15 (15) - Abdominal abscess: 13 (13) - Abdominal infection: 15 (15) - Burst abdomen: 8 (8) - Peritonitis: 6 (6) - Sepsis: 6 (6) - Urinary retention: 4 (4) - Renal failure: 7 (7) - Urinary tract infections: 8 (8)	- Oxaliplatin was associated with a higher postoperative complication rate compared with Mitomycin-C - OS was not different between 2 groups.
Pintado et al. (66)	- Population: 96 - Age: 60.7 (±9.7) - Male: 48 (50%) - BMI: NR	- Ovarian cancer: 18 (18.8) - Colorectal cancer: 43 (44.8%) - Gastric cancer: 33 (34.4%) - Pseudomyxoma: 2 (2.1)	5 (1.2–10.7)	- Transfusion of blood products during ICU stay - Bleeding complications - ICU stays - Hospital stays - ICU mortality - Hospital mortality	- Hematological complications: 77.1% - Leukopenia (66.7%), anemia (8.3%), coagulopathy (22.9%) - The median ICU stay: 5 (4.0–5.0) days - The ICU mortality rate: 1.0%	- 77.1% of patients developed hematological complications during the postoperative period; most complications were not severe and resolved spontaneously, without affecting mortality or hospital stay. - Only the development of anemia was associated with a longer ICU stay and more transfusions of blood products.
Simbert et al. (124)	- Population: 100 - Age: 54.5 (21.2–81.2) - Male: 38 - BMI: 26.6 (18.2–46.4)	- Pseudomyxomatous peritonei: 34 - Appendiceal cancer without PMP: 12 - Peritoneal mesothelioma: 3 - Colorectal: 46 - Ovarian: 4	5 (0–39)	Association between risk factors and infection outcomes	- Infection: 43 (43.0%) infections at surgical site (27), respiratory tract (9), urinary tract (11), clostridium difficile (2), post-operative sepsis (15) - Infection onset: within 7 days post-operatively - Median length of hospitalisation: 19 days for patients with infection, compared to 8 days for those without - Deaths at 60 days: 0	Importance of early infective complications in these groups of patients and benefits of monitoring in the Higher Risk subgroup (including those with small bowel resection and increased number of resected viscera).
Lee et al. (125)	- Population: 124 - Age: No neutropenia: 50.7 (±14.4) Mild neutropenia: 53.3 (±12.5) Severe neutropenia: 59.4 (±10.6) - Male: 51 - BMI: No neutropenia: 23.7 ± 4 Mild neutropenia: 22.6 (±3.8) Severe neutropenia: 23.1 (±2.9)	Colorectal cancer	No neutropenia: PCI < 10: 25 (54.3) 10 ≤ PCI < 20: 14 (30.4) 20 ≤ PCI < 30: 7 (15.2) Mild neutropenia: PCI < 10: 22 (73.3) 10 ≤ PCI < 20: 6 (20) 20 ≤ PCI < 30: 2 (6.7) Severe neutropenia: PCI < 10: 24 (50) 10 ≤ PCI < 20: 15 (31.2) 20 ≤ PCI < 30: 9 (18.8)	Evaluation of the clinical manifestations and impact of Mitomycin-C-induced neutropenia	- Mild neutropenia: 30 (24.2%) - Severe neutropenia: 48 (38.7%) - Severe neutropenia developed earlier than mild neutropenia (6.9 vs. 10.4 days) - Severe neutropenia lasted longer than mild neutropenia (4.6 vs. 2.5 days) - The rate of major postoperative complications was higher in the severe neutropenia group than in the no and mild neutropenia groups.	- Severe neutropenia starts earlier and lasts longer than mild neutropenia using a Mitomycin-C triple method - Incidence of a higher rate of major postoperative complications in patients with severe neutropenia
Bisgin et al. (100)	- Population: 362 - Age: 54.3 (±13.7) - Male: 99 (27.3) - BMI: 28.4 (±14.2)	- Ovarian: 173 (37.8) - Colorectal: 131 (36.2) - Appendix: 43 (11.9) - Gastric: 21 (5.8) - Peritoneal malignant mesothelioma: 15 (4.1) - Other: 15 (4.1)	11.6 ± 5.6	Determine the risk factors associated with GI anastomotic leak	GI anastomotic leak: 38 (10.5%)	Patient-related factors such as smoking, comorbidity, and pre-operative nutritional status had an impact on anastomotic complications.

DVT, deep vein thrombosis; PE, pulmonary embolism; CVC, central venous catheter; TIA, transient ischemic attack; MI, myocardial infarction; NCI-CTCAE, National Cancer Institute-Common Terminology Criteria for Adverse Events; OS, overall survival; AKI, acute kidney injury; CKD, chronic kidney disease; ICU, intensive care unit; GI, gastrointestinal.

In summary, although CRS + HIPEC may offer an effective surgical treatment option for cancer patients with peritoneal cavity metastases, it is essential to note that it comes with a range of associated complications. Therefore, the multidisciplinary care of patients following CRS + HIPEC involves coordinated efforts from a team of healthcare professionals to manage complex postoperative needs and optimize recovery. This team typically includes surgical oncologists, medical oncologists, anesthesiologists, intensive care specialists, nurses, nutritionists, physical therapists, and social workers, each contributing specific expertise. Post CRS + HIPEC, patients may face challenges like fluid imbalances, infection risks, nutritional deficits, and delayed wound healing, all of which require vigilant monitoring and tailored interventions. Effective multidisciplinary care enhances recovery, reduces complications, and addresses the physical and emotional well-being of patients, fostering a comprehensive approach to their long-term health and quality of life.
